# Supplementary material for: Potential Benefits of Continuous Glucose Monitoring for Predicting Vascular Outcomes in Type 2 Diabetes: A Rapid Review of Primary Research
Source: Healthcare (Basel). 2024 Aug 4;12(15):1542. doi: 10.3390/healthcare12151542 (PMC11312427; doi:10.3390/healthcare12151542)
Supplement: Supplementary file 1 [file healthcare-12-01542-s001.zip › Supplementary Table S1-Full results of database searches for the rapid review.pdf]

Supplementary Table S1. Full results of database searches for the rapid review

The Cochrane Library, Medline (via EBSCOhost), and Scopus databases were searched on 15 December 2023 to identify relevant studies. Full record of database searches:

MEDLINE (via EBSCOhost interface)

Search #	Concept	Search terms	Results
#1	Population (Patients with type 2 diabetes mellitus)	“diabetes mellitus” OR “non-insulin-dependent diabetes” OR “type 2 diabetes”	570,554
#2	Intervention (Continuous blood glucose monitoring)	“continuous glucose monitoring” OR CGM OR “flash glucose monitoring” OR “flash GM” OR “intermittently scanned CGM” OR isCGM OR “real-time CGM” OR ((fluctuation* OR monitor* OR self-monitor* OR variab*) N10 (“blood glucose” OR glycaemic OR “interstitial glucose” OR “plasma glucose” OR “serum glucose”))	23,528
#3	Comparison intervention (Haemoglobin A1c Testing)	“glycated h#emoglobin” OR “h#emoglobin A1c” OR HbA1c	78,231
#4	Outcomes of interest (Complications)	lesions OR “heart disease” OR “macrovascular” OR MI OR “microvascular” OR “myocardial infarction” OR nephropathy OR neuropathy OR pathology OR “peripheral artery disease” OR retinopathy OR stroke OR vascular	6,799,920
#5		#1 AND #2 AND #3 AND #4	954
	Date: 2000-	Limiter applied	865
	Language: English	Limiter applied	836

Cochrane Library

Search #	Concept	Search terms	Results
#1	Population (Patients with type 2 diabetes mellitus)	“diabetes mellitus” OR “non-insulin-dependent diabetes” OR “type 2 diabetes”	88,065
#2	Intervention (Continuous blood glucose monitoring)	“continuous glucose monitoring” OR CGM OR “flash glucose monitoring” OR “flash GM” OR “intermittently scanned CGM” OR isCGM OR “real-time CGM” OR ((fluctuation* OR monitor* OR self-monitor* OR variab*) NEAR/10 (“blood glucose” OR glycaemic OR “interstitial glucose” OR “plasma glucose” OR “serum glucose”))	9,628

#3	Comparison intervention (Haemoglobin A1c Testing)	“glycated haemoglobin” OR “haemoglobin A1c” OR “HbA1c”	32,097
#4	Outcomes of interest (Complications)	lesions OR “heart disease” OR “macrovascular” OR MI OR “microvascular” OR “myocardial infarction” OR nephropathy OR neuropathy OR pathology OR “peripheral artery disease” OR retinopathy OR stroke OR vascular	311,538
#5		#1 AND #2 AND #3 AND #4	467
	Date: 2000-	Limiter applied	452

Scopus

Search #	Concept	Search terms	Results
#1	Population (Patients with type 2 diabetes mellitus)	“diabetes mellitus” OR “non-insulin-dependent diabetes” OR “type 2 diabetes”	984,849
#2	Intervention (Continuous blood glucose monitoring)	“continuous glucose monitoring” OR CGM OR “flash glucose monitoring” OR “flash GM” OR “intermittently scanned CGM” OR isCGM OR “real-time CGM” OR ((fluctuation* OR monitor* OR self-monitor* OR variab*) W/10 (“blood glucose” OR glycaemic OR “interstitial glucose” OR “plasma glucose” OR “serum glucose”))	47,465
#3	Comparison intervention (Haemoglobin A1c Testing)	“glycated haemoglobin” OR “haemoglobin A1c” OR “HbA1c”	129,249
#4	Outcomes of interest (Complications)	lesions OR “heart disease” OR “macrovascular” OR MI OR “microvascular” OR “myocardial infarction” OR nephropathy OR neuropathy OR pathology OR “peripheral artery disease” OR retinopathy OR stroke OR vascular	5,757,628
#5		#1 AND #2 AND #3 AND #4	2,751
	Date: 2000-	Limiter applied	2,649
	Language: English	Limiter applied	2,492
	Document Type: Article, Review	Limiter applied	2,256
