# Supplementary material for: Potential Benefits of Continuous Glucose Monitoring for Predicting Vascular Outcomes in Type 2 Diabetes: A Rapid Review of Primary Research
Source: Healthcare (Basel). 2024 Aug 4;12(15):1542. doi: 10.3390/healthcare12151542 (PMC11312427; doi:10.3390/healthcare12151542)
Supplement: Supplementary file 1 [file healthcare-12-01542-s001.zip › Supplementary Table S3-Risk of Bias assessment for the study by Ajjan et al (2023).pdf]

Supplementary Table S3. Risk of Bias assessment for the study by Ajjan et al. (2023) based on revised Cochrane risk-of-bias tool for randomized trials (RoB 2).

Domain 1: Risk of bias arising from the randomization process

Signalling questions	Comments	Response options
**1.1 Was the allocation sequence random?**		Y
**1.2 Was the allocation sequence concealed until participants were enrolled and assigned to interventions?**		N/A
**1.3 Did baseline differences between intervention groups suggest a problem with the randomization process?**		N
**Risk-of-bias judgement**		Low
Optional: What is the predicted direction of bias arising from the randomization process?		Unpredictable

Domain 2: Risk of bias due to deviations from the intended interventions (*effect of assignment to intervention*)

Signalling questions	Comments	Response options
**2.1. Were participants aware of their assigned intervention during the trial?**		PY
**2.2. Were carers and people delivering the interventions aware of participants' assigned intervention during the trial?**		PY
**2.3. If Y/PY/NI to 2.1 or 2.2: Were there deviations from the intended intervention that arose because of the trial context?**		N
**2.4 If Y/PY to 2.3: Were these deviations likely to have affected the outcome?**		NA
**2.5. If Y/PY/NI to 2.4: Were these deviations from intended intervention balanced between groups?**		NA
**2.6 Was an appropriate analysis used to estimate the effect of assignment to intervention?**		Y
**2.7 If N/PN/NI to 2.6: Was there potential for a substantial impact (on the result) of the failure to analyse participants in the group to which they were randomized?**		NA
**Risk-of-bias judgement**		Low
Optional: What is the predicted direction of bias due to deviations from intended interventions?		Towards null

Domain 2: Risk of bias due to deviations from the intended interventions (*effect of adhering to intervention*)

Signalling questions	Comments	Response options
**2.1. Were participants aware of their assigned intervention during the trial?**		PY
**2.2. Were carers and people delivering the interventions aware of participants' assigned intervention during the trial?**		PY
**2.3. [If applicable:] If Y/PY/NI to 2.1 or 2.2: Were important non-protocol interventions balanced across intervention groups?**		PY
**2.4. [If applicable:] Were there failures in implementing the intervention that could have affected the outcome?**		PN
**2.5. [If applicable:] Was there non-adherence to the assigned intervention regimen that could have affected participants' outcomes?**		NI
**2.6. If N/PN/NI to 2.3, or Y/PY/NI to 2.4 or 2.5: Was an appropriate analysis used to estimate the effect of adhering to the intervention?**		NI
**Risk-of-bias judgement**		Low
Optional: What is the predicted direction of bias due to deviations from intended interventions?		Unpredictable

Domain 3: Missing outcome data

Signalling questions	Comments	Response options
**3.1 Were data for this outcome available for all, or nearly all, participants randomized?**		PY
**3.2 If N/PN/NI to 3.1: Is there evidence that the result was not biased by missing outcome data?**		NA
**3.3 If N/PN to 3.2: Could missingness in the outcome depend on its true value?**		NA
**3.4 If Y/PY/NI to 3.3: Is it likely that missingness in the outcome depended on its true value?**		NA
**Risk-of-bias judgement**		Low
Optional: What is the predicted direction of bias due to missing outcome data?		Unpredictable

Domain 4: Risk of bias in measurement of the outcome

Signalling questions	Comments	Response options
**4.1 Was the method of measuring the outcome inappropriate?**		N
**4.2 Could measurement or ascertainment of the outcome have differed between intervention groups?**		PN
**4.3 If N/PN/NI to 4.1 and 4.2: Were outcome assessors aware of the intervention received by study participants?**		PY
**4.4 If Y/PY/NI to 4.3: Could assessment of the outcome have been influenced by knowledge of intervention received?**		PN
**4.5 If Y/PY/NI to 4.4: Is it likely that assessment of the outcome was influenced by knowledge of intervention received?**		PN
**Risk-of-bias judgement**		Some concerns
Optional: What is the predicted direction of bias in measurement of the outcome?		Unpredictable

Domain 5: Risk of bias in selection of the reported result

Signalling questions	Comments	Response options
**5.1 Were the data that produced this result analysed in accordance with a pre-specified analysis plan that was finalized before unblinded outcome data were available for analysis?**		PY
**Is the numerical result being assessed likely to have been selected, on the basis of the results, from...**		
**5.2. ... multiple eligible outcome measurements (e.g. scales, definitions, time points) within the outcome domain?**		N
**5.3 ... multiple eligible analyses of the data?**		PN
**Risk-of-bias judgement**		Low
Optional: What is the predicted direction of bias due to selection of the reported result?		Away from null

Overall risk of bias

**Risk-of-bias judgement**		Low
Optional: What is the overall predicted direction of bias for this outcome?		Away from null

This work is licensed under a Creative Commons Attribution-NonCommercial-NoDerivatives 4.0 International License.
